# Metabolic adaptations to hypoxia in the neonatal mouse forebrain can occur independently of the transporters *SLC7A5* and *SLC3A2*

**DOI:** 10.1038/s41598-021-88757-9

**Published:** 2021-04-27

**Authors:** Eamon Fitzgerald, Jennie Roberts, Daniel A. Tennant, James P. Boardman, Amanda J. Drake

**Affiliations:** 1 University/British Heart Foundation Centre for Cardiovascular Science, University of Edinburgh, The Queen’s Medical Research Institute, 47 Little France Crescent, Edinburgh, EH16 4TJ UK; 2 Institute of Metabolism and Systems Research, College of Medical and Dental Sciences, University of Birmingham, Birmingham, UK; 3 MRC Centre for Reproductive Health, University of Edinburgh, The Queen’s Medical Research Institute, 47 Little France Crescent, Edinburgh, EH16 4TJ UK; 4 Centre for Clinical Brain Sciences, University of Edinburgh, Chancellor’s Building, 49 Little France Crescent, Edinburgh, EH16 4SB UK

**Keywords:** Developmental biology, Neuroscience

## Abstract

Neonatal encephalopathy due to hypoxia–ischemia is associated with adverse neurodevelopmental effects. The involvement of branched chain amino acids (BCAAs) in this is largely unexplored. Transport of BCAAs at the plasma membrane is facilitated by SLC7A5/SLC3A2, which increase with hypoxia. We hypothesized that hypoxia would alter BCAA transport and metabolism in the neonatal brain. We investigated this using an organotypic forebrain slice culture model with, the SLC7A5/SLC3A2 inhibitor, 2-Amino-2-norbornanecarboxylic acid (BCH) under normoxic or hypoxic conditions. We subsequently analysed the metabolome and candidate gene expression. Hypoxia was associated with increased expression of *SLC7A5* and *SLC3A2* and an increased tissue abundance of BCAAs. Incubation of slices with ^13^C-leucine confirmed that this was due to increased cellular uptake. BCH had little effect on metabolite abundance under normoxic or hypoxic conditions. This suggests hypoxia drives increased cellular uptake of BCAAs in the neonatal mouse forebrain, and membrane mediated transport through *SLC7A5* and *SLC3A2* is not essential for this process. This indicates mechanisms exist to generate the compounds required to maintain essential metabolism in the absence of external nutrient supply. Moreover, excess BCAAs have been associated with developmental delay, providing an unexplored mechanism of hypoxia mediated pathogenesis in the developing forebrain.

## Introduction

Neonatal encephalopathy due to hypoxia–ischemia (HIE) can occur in both term and preterm infants^1^, and is associated with adverse long-term effects on neurodevelopment including an increased risk of learning difficulties, cerebral palsy and epilepsy^2,3^. Studies suggest that approximately 55% of infants with HIE have neurological impairment at 6–7 years of age, and reduced cognitive scores have also been reported in individuals who required resuscitation during the neonatal period but who did not fulfil the criteria for HIE^4^, indicating that transient hypoxia may also have persistent effects. Therapeutic hypothermia reduces the risk of mortality or major neurodevelopmental disability following HIE but is only effective if used within a short postnatal time period in term infants^5–8^; therefore there is a need to develop adjunctive therapies to further reduce the burden of disability. Cerebral oxygenation and metabolic disturbances (as measured by cytochrome-c-oxidase using near infrared spectroscopy and magnetic resonance spectroscopy) are well-characterised in infants with HIE and are positively correlated with neurodevelopmental outcome^9–11^. Moreover, altered motor skills^12^, memory and learning^13,14^ have been reported in rodent models of hypoxia^15^. An improved understanding of the mechanisms by which perinatal hypoxia leads to long term damage in the developing brain could facilitate the development of novel therapeutics for both term and preterm infants.

In many peripheral tissues, carbons from the branched chain amino acids (BCAAs; isoleucine, leucine and valine) are used to supply the TCA cycle, and BCAA oxidation occurs rapidly in most tissues under normal conditions^16^. The neonatal brain has a considerable energy requirement and utilises glucose, lactate and ketone bodies as sources of fuel under normal conditions although relatively little is known about the contribution of BCAA metabolism to cerebral energetics^17–19^. Studies in neonatal rodents and pigs, showing increases in the BCAAs in plasma and brain with hypoxia, suggest that BCAAs may be important in the metabolic adaptations to hypoxia in the perinatal period^20–22^. Further evidence for the potential importance of BCAAs in the hypoxia response comes from studies showing that hypoxia stimulates BCAA catabolism in a glioblastoma cell line^23^ and BCAAs are critical for cellular respiration in pancreatic ductal adenocarcinoma—a disease associated with tissue hypoxia^24,25^. Interestingly, BCAA excess in the neonatal period, as seen in maple syrup urine disease (MSUD), is also associated with developmental delay^26^. Transport of BCAAs at the plasma membrane is facilitated by SLC7A5 (solute carrier 7A5), which acts in a heterodimer with SLC3A2^27^. *SLC7A5* and *SLC3A2* mRNA are upregulated in various cell types during hypoxia in a HIF2α (Hypoxia Inducible Factor 2α) dependent manner^28–30^ suggesting that both BCAA transport and metabolism are altered under conditions of metabolic stress.

*SLC7A5* and *SLC3A2* are primarily expressed in endothelial cells in the neonatal brain^31^ and their importance is supported by studies showing that deletion of *SLC7A5* in endothelial cells results in deficits in social behaviour in mice, a phenotype that is rescued by intracerebral administration of the BCAAs leucine and isoleucine^32^. Moreover, mutations in *SLC7A5* and *SLC3A2* are causative of autism spectrum disorder^32,33^. Although there is an assumption that BCAAs can be transported rapidly in and out of cells, this is not well understood^34^.

In this study we investigated the hypothesis that hypoxia leads to changes in BCAA transport and metabolism in the neonatal forebrain using an organotypic forebrain slice culture model. BCH had little effect on the abundance of metabolites in forebrain tissue under normoxic or hypoxic conditions suggesting that robust alternative mechanisms regulate of tissue BCAA levels in the absence of SLC7A5/SLC3A2-mediated transport.

## Results

### Hypoxia affects gene expression and leads to alterations in intra- and extracellular BCAA levels

**Figure 1:**
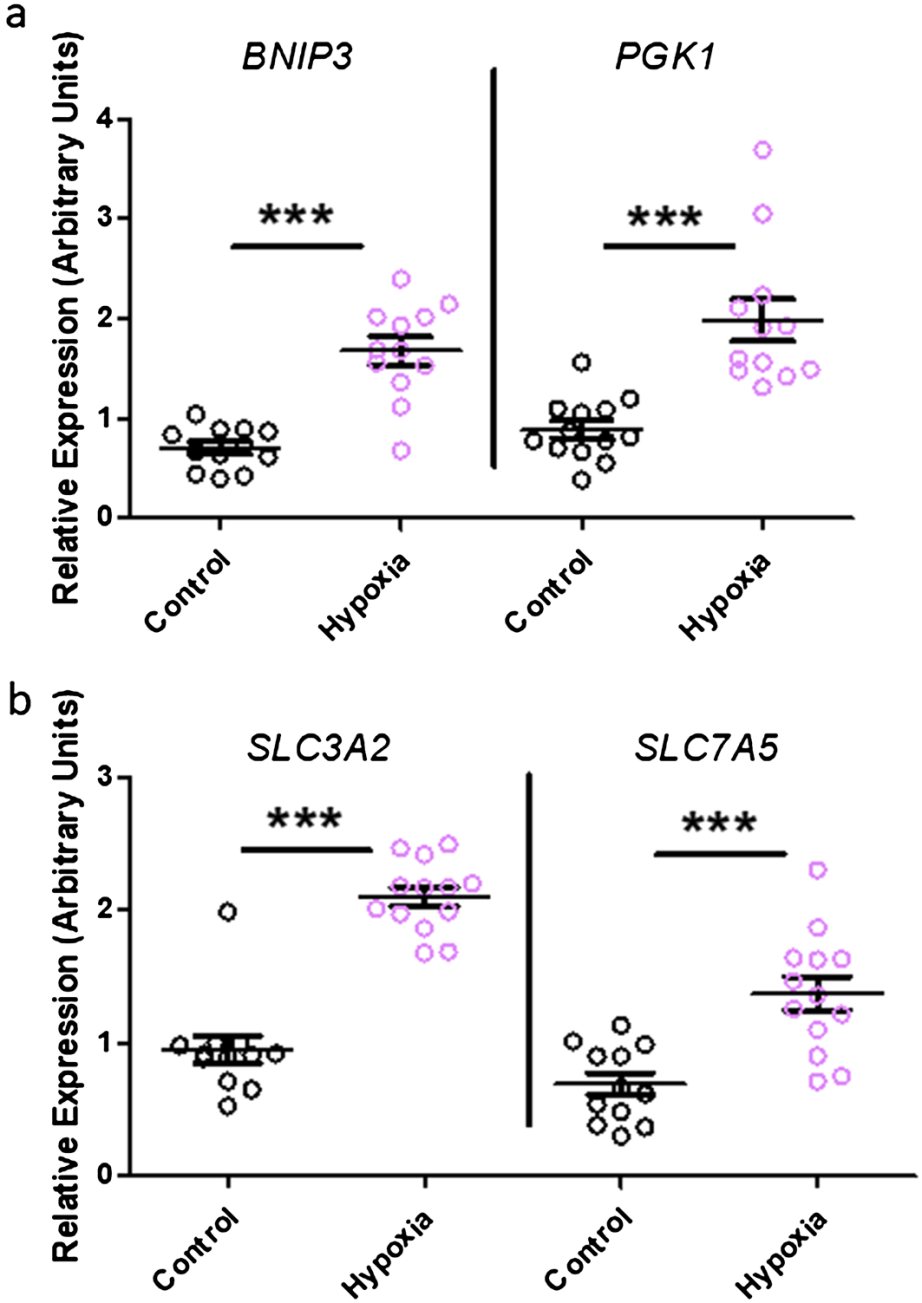
Candidate gene expression following control (black) and hypoxia (pink) conditions in the forebrain slice culture model. (**a**) Hypoxia resulted in an increased expression of the classic hypoxia response genes, BNIP3 and PGK1. (**b**) Hypoxia also resulted in an increased expression of SLC3A2 and SLC7A5. *** indicates p < 0.001, comparisons were made using an independent t-test followed by a Bonferroni p-value adjustment for multiple comparisons. Expression was normalised to TBP. Error bars indicate standard error.

Exposure to 1% oxygen for 24 h resulted in increased mRNA expression of the classic hypoxia response genes *BNIP3* (*BCL2 and adenovirus E1B 19-kDa-interacting protein 3*) (p = 4.60E−04, df = 20) and *PGK1* (*Phosphoglycerate Kinase 1*) (p = 7.88E−05, df = 22) in the forebrain slice cultures (Fig. 1a), indicating a physiologically relevant response to hypoxia. Hypoxia was also associated with an increase in the mRNA expression of the BCAA transporters *SLC3A2* (p = 4.21E−09, df = 23) and *SLC7A5* (p = 9.38E−05, df = 24) (Fig. 1b).

**Figure 2:**
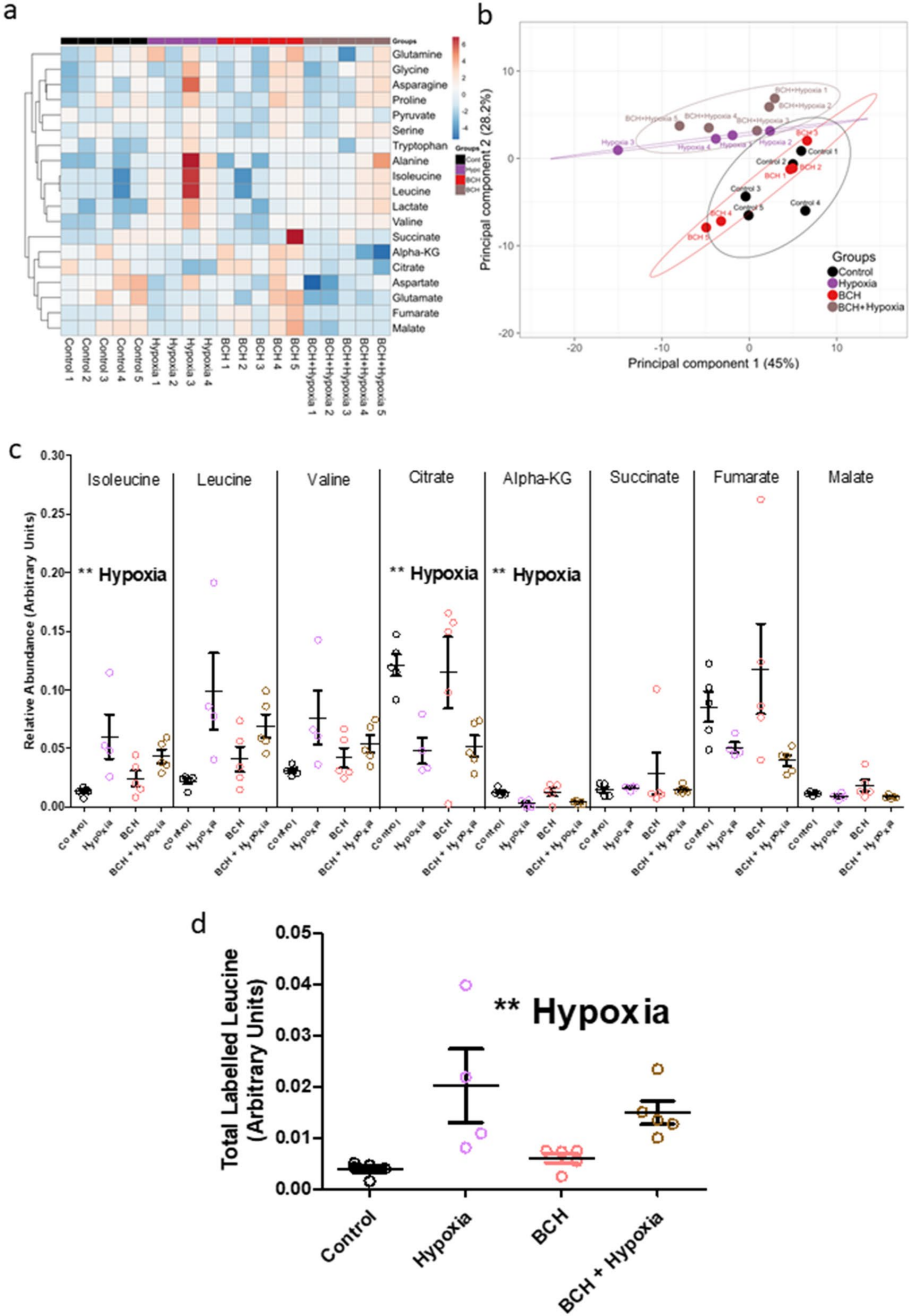
Measurement of candidate intracellular metabolites in forebrain slices of control (black), BCH (pink), hypoxia (red) and BCH + hypoxia (gold) groups. (**a**) Heatmap of all metabolites clustered with respect to fold change from the control mean (red indicates an increase, blue indicates a decrease). The 4 groups are indicated on the y-axis with individual samples identified. Metabolites are labelled on the y-axis and are clustered by Euclidian distance. (**b**) A PCA plot describes the variance among all the metabolites in the 2 principal components which explain the most variance (principal component 1 and 2 describe 45% and 28%, respectively). (**c**) There were significant main effects of hypoxia to increase the abundance of isoleucine and to decrease the abundance of citrate and alpha-ketoglutarate. There were no significant main effects of BCH or interaction effects. (**d**) Following incubation with 13C-leucine, there was an increase in intracellular 13C-leucine following hypoxia (p = 0.0016, f = 14.87), with no effect of BCH (p = 0.642, f = 0.22) and no interaction (p = 0.276, f = 1.28). ** indicates p < 0.003 which was the adjusted threshold for statistical significance. Statistical comparisons were made using a 2-way ANOVA using a Bonferroni adjusted p-value. Metabolite abundance was normalised to the spiked in standard 6-Glutaric acid. Error bars indicate standard error. A and B were generated using ClustVis version 1 (http://biit.cs.ut.ee/clustvis/).

We next assessed the effect of hypoxia on the levels of BCAAs and a number of TCA cycle-associated metabolites in the cultured forebrain slices (Fig. 2a). PCA confirmed that the slice culture samples clustered according to hypoxia status (Fig. 2b). Exposure to hypoxia was associated with an increase in the abundance of isoleucine but changes in leucine and valine did not survive correction for multiple comparisons (Fig. 2c and Table 1). Incubation of cells with ^13^C-leucine confirmed that hypoxia caused an increase in the cellular uptake of ^13^C-leucine (Fig. 2d). Hypoxia was also associated with decreases in the abundance of intracellular citrate and alpha-ketoglutarate and with increased abundance of lactate and glutamate but there were no changes in the abundance of other TCA cycle-associated metabolites including pyruvate, succinate, fumarate or malate (Fig. 2c and Table 1).

**Table 1:** List of intracellular metabolites examined along with their unadjusted p-value and F-statistic for main effects of hypoxia and BCH, as well as an interaction effect.

Metabolite	Unadjusted p-value (F-statistic)		
	Hypoxia	BCH	Interaction
Alanine	0.015 (7.507) ↑	0.499 (0.479)	0.55 (0.374)
Alpha-KG	**0.001 (18.933) ↓	0.81 (0.06)	0.791 (0.073)
Asparagine	0.196 (1.829)	0.659 (0.203)	0.385 (0.801)
Aspartate	0.029 (5.788) ↓	0.135 (2.492)	0.725 (0.128)
Citrate	**0.002 (13.906) ↓	0.954 (0.003)	0.775 (0.085)
Fumarate	0.04 (5.073) ↓	0.265 (1.34)	0.189 (1.894)
Glutamate	**0.002 (14.881) ↓	0.595 (0.295)	0.927 (0.009)
Glutamine	0.624 (0.251)	0.629 (0.243)	0.362 (0.884)
Glycine	0.512 (0.451)	0.922 (0.01)	0.509 (0.458)
Isoleucine	**0.003 (12.937) ↑	0.757 (0.099)	0.145 (2.365)
Lactate	***0.0001 (26.062) ↑	0.751 (0.105)	0.401 (0.748)
Leucine	0.004 (11.415) ↑	0.732 (0.122)	0.14 (2.427)
Malate	0.021 (6.674) ↓	0.637 (0.232)	0.334 (0.996)
Proline	0.933 (0.007)	0.876 (0.025)	0.469 (0.552)
Pyruvate	0.809 (0.061)	0.595 (0.295)	0.45 (0.601)
Serine	0.245 (1.465)	0.798 (0.068)	0.884 (0.022)
Succinate	0.539 (0.395)	0.537 (0.399)	0.44 (0.629)
Tryptophan	0.122 (2.684)	0.449 (0.604)	0.086 (3.385)
Valine	0.022 (6.471) ↑	0.618 (0.259)	0.162 (2.158)

Arrows indicating direction of change are present for all comparisons with p < 0.05. Values in bold remain significant after correction for multiple comparisons.

** indicates *p* < 0.01, *** indicates *p* < 0.001.

**Figure 3:**
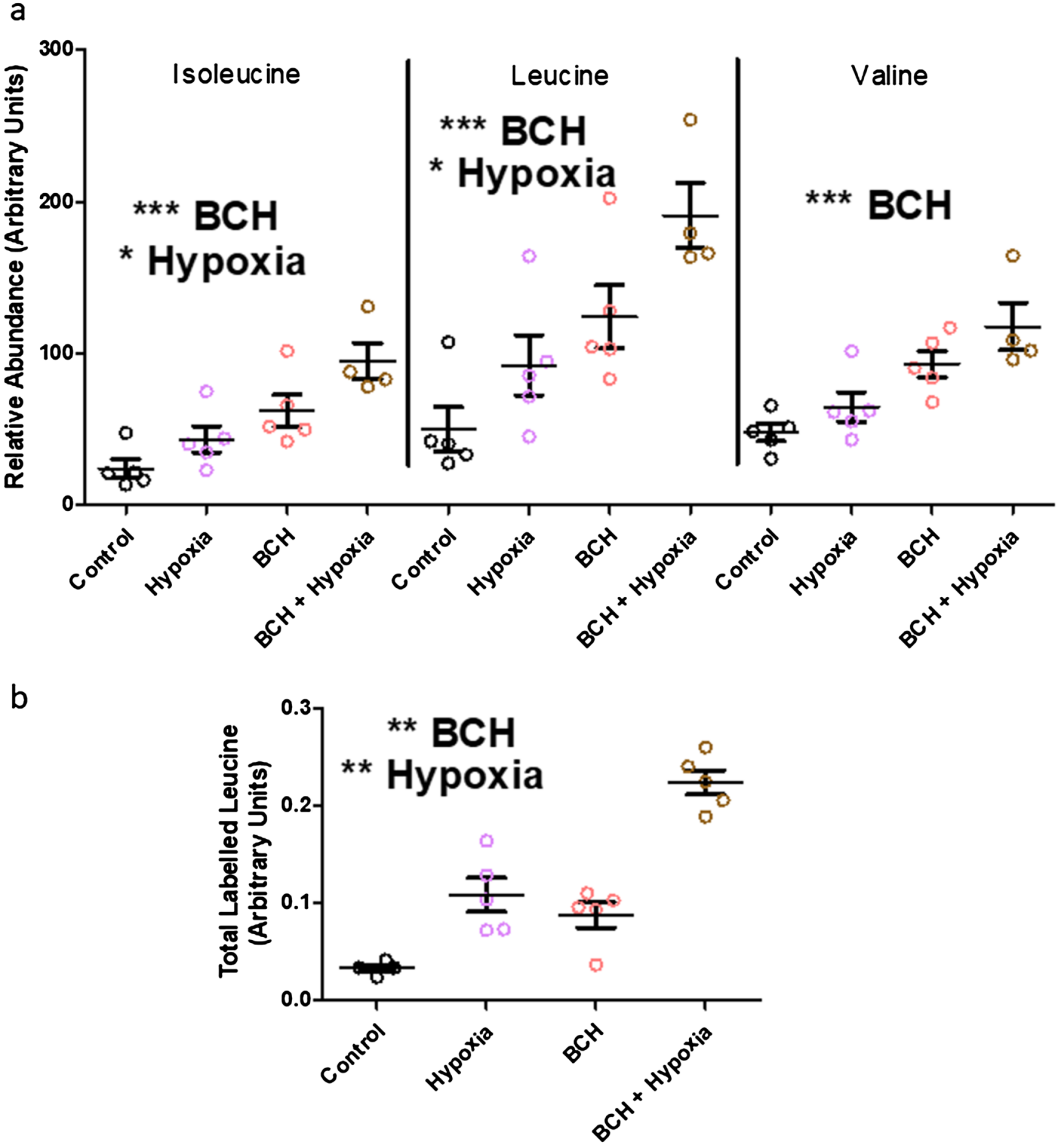
Measurement of candidate metabolites in the supernatant of forebrain slices of control (black), BCH (pink), hypoxia (red) and BCH + hypoxia (gold) groups. (**a**) There was a significant main effect of BCH to increase the abundance of isoleucine (p = 0.0002, f = 22.80), leucine (p = 0.004, f = 19.99) and valine (p = 0.0002, f = 24.28). There was also a significant main effect of hypoxia to increase isoleucine (p = 0.014, f = 7.68) and leucine (p = 0.013, f = 7.91), but not valine (p = 0.056, f = 4.31). There was no evidence for an interaction effect for any of the BCAAs (isoleucine-p = 0.489, f = 0.50; leucine-p = 0.54, f = 0.40; valine-p = 0.70, f = 0.15). (**b**) 13C-leucine concentrations in the supernatant were higher with hypoxia (p = 0.009, f = 9.02) and BCH (p = 0.004, f = 11.62), with no interaction effects (p = 0.715, f = 0.14). ** indicates p < 0.01. *** indicates p < 0.001, adjusted threshold for statistical significance was p < 0.003. Statistical comparisons were made using a 2-way ANOVA. Metabolite abundance was normalised to the spiked in standard 6-Glutaric acid. Error bars indicate standard error.

In the supernatant, hypoxia was associated with higher levels of leucine and isoleucine (Fig. 3a) and analysis of the abundance of labelled leucine confirmed that hypoxia resulted in higher levels of labelled leucine in the supernatant in comparison to control (Fig. 3b).

### Hypoxia affects the expression of genes involved in BCAA transport in forebrain slices

**Figure 4:**
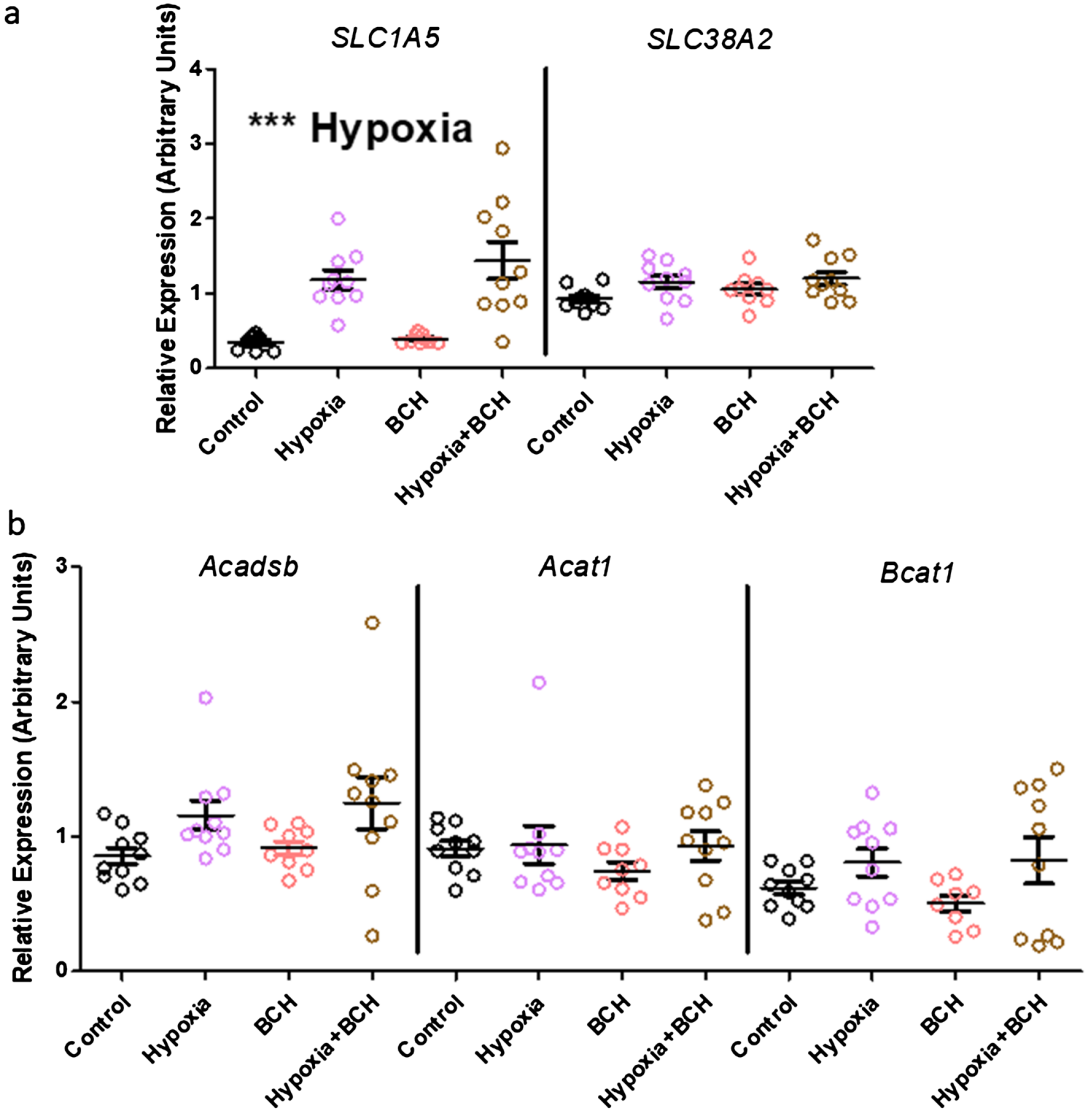
Candidate expression of genes involved in BCAA transport (**a**) and BCAA catabolism (**b**) for control (black), BCH (pink), hypoxia (red) and BCH + hypoxia (gold) groups. (**a**) Hypoxia was associated with an increase in the expression of SLC1A5 (p = 1.53E−7, f = 42.70) but not SLC38A2 (p = 0.017, f = 6.29). There were no main effects of BCH (SLC1A5-p = 0.30, f = 1.09; SLC38A2-p = 0.25, f = 1.37) or interaction effects (SLC1A5-p = 0.47, f = 0.54; SLC38A2-p = 0.61, f = 0.26). (**b**) There were no significant main effects of hypoxia or BCH on the expression of Acadsb (hypoxia-p = 0.01, f = 6.95; BCH-p = 0.53, f = 0.39), Acat1 (hypoxia-p = 0.30, f = 1.13; BCHp = 0.40, f = 0.74) or Bcat1 (hypoxia-p = 0.04, f = 4.76; BCH-p = 0.75, f = 0.10) and no interaction effects (Acadsb-p = 0.89, f = 0.02; Acat1-p = 0.43, f = 0.62; Bcat1-p = 0.65, f = 0.21). p < 0.004 was the adjusted threshold for statistical significance, *** indicates p < 0.001. Statistical comparisons were made using a 2-way ANOVA using a Bonferroni adjusted p-value. Expression was normalised to TBP. Error bars indicate standard error.

Analysis of the expression of potential compensatory BCAA transporters revealed increased expression of *SLC1A5* during hypoxia (Fig. 4a). There were no changes in expression of the transporter *SLC38A2* or in candidate genes involved in BCAA catabolism (*Acadsb* (*Short/branched chain acyl-CoA dehydrogenase*), *Acat1* (*Acetyl-CoA Acetyltransferase 1*), *Bcat1* (*Branched Chain Amino Acid Transaminase 1*)) (Fig. 4b). There was also an increase in expression of the pro-apoptotic gene, *Bax* (Table 2). Details of all candidate genes tested and relevant statistical information are present in Table 2.

**Table 2:** List of candidate gene expression effects including their unadjusted p-value and F-statistic for main effects of hypoxia and BCH, as well as an interaction effect.

Gene	Unadjusted p-value (F-statistic)		
	Hypoxia	BCH	Interaction
**Enzymes involved in BCAA catabolism**			
*Acadsb*	0.012 (6.95) ↑	0.538 (0.388)	0.891 (0.019)
*Acat1*	0.295 (1.13)	0.397 (0.737)	0.437 (0.617)
*Bcat1*	0.036 (4.759) ↑	0.751 (0.102)	0.653 (0.205)
*Ivd*	0.224 (1.531)	0.945 (0.005)	0.974 (0.001)
*Hmgcll1*	0.063 (3.68)	0.516 (0.43)	0.67 (0.185)
**Apoptosis related genes**			
*Bax*	***0.0001 (18.719) ↑	0.108 (2.721)	0.381 (0.787)
*Bcl2*	0.309 (1.066)	0.225 (1.527)	0.92 (0.01)
*Bad*	0.352 (0.889)	0.103 (2.802)	0.835 (0.044)
**Proliferation related genes**			
*Ki67*	0.473 (0.525)	0.18 (1.875)	0.554 (0.357)
*PCNA*	0.01 (7.353) ↑	0.121 (2.529)	0.961 (0.002)
**Amino acid transporters**			
*SLC1A5*	***1.53E−7 (42.704) ↑	0.304 (1.089)	0.467 (0.54)
*SLC38A2*	0.017 (6.289) ↑	0.25 (1.371)	0.614 (0.259)

Arrows indicating direction of change are present for all comparisons with p < 0.05. Values in bold remain significant after correction for multiple comparisons.

*** indicates *p* < 0.001.

### Inhibition of SLC7A5/SLC3A2 transport results in alterations in extracellular metabolites

Considering the potential role of SLC7A5/SLC3A2 in altering BCAA uptake in hypoxia, we also investigated the effects of SLC7A5/SLC3A2 inhibition on gene expression and the abundance of BCAAs and related metabolites using BCH; a well characterised SLC7A5/SLC3A2 inhibitor^27^. In the cultured forebrain slices, there was no effect of BCH on the abundance of intracellular BCAAs or any of the other metabolites measured (Fig. 2c,d). In contrast, BCH was associated with higher levels of isoleucine, leucine and valine in the supernatant (Fig. 3a) and analysis of the abundance of labelled leucine confirmed that incubation with BCH resulted in higher levels of labelled leucine in the supernatant in comparison to control (Fig. 3b). BCH exposure was not associated with changes in the mRNA expression of genes involved in BCAA catabolism or alternative BCAA transporters. Details of all candidate genes tested, and relevant statistical information are presented in Table 2.

## Discussion

In situations of hypoxia, biochemical responses are necessary to offset the decrease in energy supply from mitochondrial respiration^35^ and data suggest that hypoxia in peripheral tissues results in an increase in intracellular BCAAs through the action of the SLC7A5/SLC3A2 complex^28^. We hypothesised that exposure to hypoxia would be associated with an increase in BCAA uptake into cells of the neonatal forebrain. Using an organotypic forebrain slice culture system we showed that exposure to hypoxia was associated with an increase in the tissue abundance of isoleucine and leucine. Incubation with ^13^C-leucine confirmed that at least in part, this is due to increased cellular uptake.

Hypoxia was associated with an increase in the mRNA expression of the BCAA transporters *SLC7A5* and *SLC3A2* in the forebrain. However, a number of other amino acid transporters including SLC1A5 have varying levels of affinity for BCAAs^36^ and SLC38A2 can facilitate efficient BCAA uptake through SLC7A5/SLC3A2^37^. The mRNA expression of the amino acid transporter *SLC1A5*, which can also transport BCAAs, was increased by hypoxia, suggesting activation of a general cellular program to increase intracellular amino acids during hypoxic events. To begin to explore the role of SLC7A5/SLC3A2 further, we used BCH to study the effects of SLC7A5/SLC3A2 inhibition on metabolite abundance. Whilst the higher levels of all three BCAAs in the supernatant with BCH exposure indicates functional inhibition of BCAA cellular uptake by SLC7A5/SLC3A2, BCH had little effect on the abundance of metabolites in forebrain tissue under normoxic or hypoxic conditions. This suggests robust alternative mechanisms exist to regulate tissue BCAA levels in the absence of SLC7A5/SLC3A2-mediated transport.

Although BCAAs can be oxidised into intermediates which can enter the TCA cycle, tissue hypoxia was associated with decreases in the tissue abundance of the TCA cycle metabolites citrate and alpha-ketoglutarate. These decreases, along with an accumulation of lactate, are in line with the well characterised shift to glycolysis during hypoxia in the neonatal brain which has been described in models of neonatal hypoxia–ischemia^38,39^ and in human neonates following HIE^40^. BCAA nitrogen can also be used in the generation of glutamate and we observed an increase in the tissue levels of glutamate with hypoxia. Glutamate is the major excitatory neurotransmitter in the brain and plays a key role in the damage associated with neonatal HIE^41^.

Despite the hypoxia-induced increase in intracellular BCAAs and evidence from the use of ^13^C-leucine that cellular uptake is increased in hypoxia, the abundance of BCAAs was also higher in the supernatant in comparison to controls. This suggests that mechanisms unrelated to external nutrient supply have been activated to supply the compounds needed to maintain essential metabolism. Possible mechanisms for this include a change in the activity of other transporters—one of particular note is ASCT2 (SLC1A5), which can antiport cellular leucine against extracellular glutamine in some circumstances^42^. Another potentially linked mechanism may be through alterations in autophagy, which is thought to be important for the maintenance of ATP production under conditions of hypoxia^35^. Autophagy is an important process in the developing brain and is strictly controlled, but may be influenced by environmental exposures^43^. Although autophagy in response to cellular stress may represent an adaptive mechanism which helps to maintain cellular homeostasis, its role in hypoxia–ischaemia is controversial^43^, with some studies suggesting that it may contribute to neuronal injury while^2,44^ others propose a protective role. Also of note is the role of SLC7A5 in transporting thyroid hormone across the plasma membrane^45,46^. This function may also drive hypoxia mediated SLC7A5 alterations.

We show hypoxia drives BCAA accumulation in both the intracellular and extracellular space. This is associated with neurodevelopmental delay in MSUD^26^. Therefore, future studies should also consider the pathogenic consequences of increased BCAAs during hypoxia and their effect on brain development.

This study had a number of important limitations. BCH, which we used to inhibit BCAA uptake, also has affinity for other large neutral amino acid transporters. Further experiments using more specific methods such as the inhibitor JPH203 or genetic manipulation of SLC7A5 are required to fully delineate its role in BCAA transport during hypoxic conditions in the neonatal brain. Furthermore, we used a single concentration of BCH (10 mM) and whilst several studies have shown this dose provides robust inhibition of SLC7A5 mediated transport^27,47,48^, future studies investigating dose–response relationships will be important for the demonstration of causation. Finally, we measured SLC7A5 and SLC3A2 mRNA levels in this study. Even though we provide evidence of a functional upregulation of BCAA transport through these transporters, future studies will need to verify this upregulation at the protein level.

In conclusion, we show that hypoxia drives increases in intracellular BCAAs in the neonatal mouse forebrain and suggest that membrane mediated transport through the transporters *SLC7A5* and *SLC3A2* may not be essential for this process. Our data showing that hypoxia associates with increased extracellular BCAAs suggest that mechanisms exist to generate the compounds required to maintain essential metabolism in the absence of external nutrient supply, which occurs under ischaemic conditions. Considering increased BCAAs are also associated with developmental delay in MSUD, this may also be a mechanism of hypoxia induced toxicity.

## Methods

### Animals

C57/BL6J/OLA (referred to hereafter as C57/BL6) mice (Harlan, UK) had ad libitum access to chow (Special Diets Services, Essex, UK) and water (lights on 07:00–19:00, temperature 22 °C). Two females and 1 male were kept per cage for mating. Dams were checked daily for new litters with postnatal day (P) 0 designated as the day of birth. All experiments were approved by The University of Edinburgh and in accordance with the UK Home Office Animals (Scientific Procedures) Act 1986 and ARRIVE guidelines. Both male and female animals were used in all experiments described. For initial candidate gene expression, metabolite and final candidate gene expression analysis n = 12, 5 and 10 per group, respectively.

### Organotypic forebrain slice culture

Organotypic forebrain slice culture was carried out in line with previous studies^49,50^. The forebrain was chosen due to well characterised patterns of injury here following neonatal hypoxia^51,52^. C57BL/6 pups were killed at P0 or P1 with an overdose of Pentobarbitone (Euthatal) administered via intraperitoneal injection at 150 mg/kg. Pups were kept on ice until whole brains were dissected into ice-cold L15 media (Thermo Fisher Scientific, UK). Next, 300 µm coronal sections were cut using a McaIlwain Tissue Chopper (Campden Instruments LTD, Loughborough). Brains were then transferred to a petri-dish of slice culture media (formula described later), where brain slices were separated using a Wild Heerbrugg 1 × objective for the Wild M8 Stereo Zoom Microscope. Slices were then transferred using a spatula onto a 30 mm, 0.4 µm hydrophilic Millicell culture insert (Merck, UK) in a 6-well plate with 1 ml/well of slice culture media. Slice culture media consisted of 25% EBSS (Thermo Fisher Scientific, UK), 67% BME media (Thermo Fisher Scientific, UK), 5% heat inactivated horse serum (Thermo Fisher Scientific, UK), 1% penicillin/streptomycin (Sigma-Aldrich, UK), 1% GlutaMAX-I supplement (Thermo Fisher Scientific, UK) and 1% D( +)-Glucose (Sigma-Aldrich, UK). Only slices from the genu of the corpus callosum to the dorsal hippocampus were cultured. All forebrain slices were kept in an incubator at 37 °C with 5% CO_2_ and ambient oxygen levels, unless otherwise stated. The same batch of slice culture media were used for all experiments to limit variation. Slices were cultured for 7 days before exposure to their respective treatment for 24 h.

For hypoxic conditions, slices were maintained in 1% oxygen and 5% CO_2_ for 24 h using a Coy in vitro chamber (Coy, Michigan). Forebrain slices and supernatant were frozen on dry ice immediately after removal from the hypoxia chamber. For culture with 2-Amino-2-norbornanecarboxylic acid (BCH) (Sigma-Aldrich, UK), BCH was reconstituted in sterile PBS. BCH was added to the slices at a concentration of 10 mM, which has previously been shown to effectively inhibit SLC7A5/SLC3A2 transport^27,47,48^. An equal volume of PBS was added concurrently to the control slices. For slices used for metabolic labelling experiments 0.12 mM ^13^C-Leucine (CK Isotopes, Leicestershire) was added to the media at the onset of hypoxia and/or BCH for 24 h.

The experimental set up therefore included four groups: (1) control (PBS + normoxia), (2) hypoxia (PBS + hypoxia) (3) SLC7A5/SLC3A2 transport inhibition (BCH + normoxia) 4) BCH + hypoxia.

### RNA extraction, reverse transcription and quantitative PCR

RNA was extracted using Qiazol (Qiagen, Manchester, UK) and the RNeasy mini kit (Qiagen, Manchester, UK), as per manufacturer’s instructions. RNA was eluted in 40 µl of nuclease free water (Qiagen, Manchester, UK) and the concentration determined using the Qubit Fluorometer 2.0 (Thermo Fisher Scientific, UK). 1 µg RNA was DNase treated with RQ1 RNase free DNase (Promega, Southampton, UK) as per manufacturer’s instructions. Reverse transcription with the Applied Biosystems RT kit (Thermo Fisher Scientific, UK) was carried out as per manufacturer’s instructions using a G-Storm Thermocycler (Akribis Scientific Limited, Cheshire). RNA was stored long term at − 80 °C. Primers for qPCR were designed using the UPL Assay Design Centre and cDNA samples were analysed in triplicate using a Roche LightCycler 480. Sample gene expression was normalised to the expression of the housekeeping gene *TBP* (*TATA-Box Binding Protein*) using relative standard curves. Primers used are listed in Table 3.

**Table 3:** List of qPCR primers used, along with their respective probes from the universal probe library.

Gene	Forward primer (5′ → 3′)	Reverse primer (5′ → 3′)	Probe
*Acadsb*	gctccagctgtggcgtat	caaggagacaagcaggttgg	66
*Acat1*	gcagctgctctggttctcat	tacggcagcatcagcaaat	34
*Bcat1*	tcattctcccaggagtgacc	ccatggtgaggtgtctctca	1
*Bax*	gtgagcggctgcttgtct	ccatcttcttccagatggtga	5
*Bcl2*	gtacctgaaccggcatctg	gctgagcagggtcttcagag	2
*Bad*	ccaccaacagtcatcatgga	cgtcctcgaaaagggctaa	25
*Ki67*	cacctggtcaccatcaagc	gcagctggatacgaatgtca	17
*Ivd*	gaaagagaaataccttcccaagc	cttttctgcttttagcttcatgg	21
*Hmgcll1*	cctaatcttcagggttttcagc	tgaagcagcaccaaaaactg	67
*PCNA*	ctagccatgggcgtgaac	gaatactagtgctaaggtgtctgcatt	41
*SLC1A5*	gcagtgttcatcgcacaacta	atgctgtggctgtgaccag	9
*SLC38A2*	caatgagatccgtgcaaaag	tgcttccaatcatcaccact	2
*TBP*	gggagaatcatggaccagaa	gatgggaattccaggagtca	97

### Metabolite extraction

Immediately following the treatment period brain slices and supernatant were frozen on dry ice and stored at − 80 °C for subsequent metabolite extraction. A single biological replicate was derived from 5 individual slices which were cultured on the same insert. Metabolites were extracted and analysed as described in Hollinshead et al.^53^. In brief, 1.2 ml of pre-chilled 0.005% butylated hydroxytoluene (LKT Labs, Minnesota) in methanol was added to samples, followed by a 1-min incubation. Next, 4 µg of D6-Glutaric acid (CDN Isotopes, Quebec; used as an internal standard) in 1.2 ml of HLPC grade water was added. Samples were vortexed before the addition of 1.2 ml of chloroform, followed by incubation under agitation for 15 min at 4 °C. Samples then underwent centrifugation at 16,000×g for 10 min at 4 °C. The aqueous phase was removed and dried to a powder using a Savant SpeedVac Concentrator (Thermo Fisher Scientific, UK).

### Gas chromatography–mass spectrometry (GC–MS)

Derivatized samples were analysed using an Agilent 7890B/5977A GC–MS (Agilent Technologies, Cheshire). First, 1 μl of sample was injected in splitless mode with helium carrier gas at a rate of 1.0 ml min^−1^. Initial oven temperature was held at 100 °C for 1 min before ramping to 170 °C at a rate of 10 °C min^−1^, followed by a ramp to 220 °C at a rate of 3 °C min^−1^ and a final ramp to 300 °C at a rate of 10 °C min^−1^ with a 5 min hold. Compound detection was carried out in single ion monitoring (SIM) mode. Total ion counts of each metabolite were normalised to the internal standard D6-Glutaric acid.

### Statistical analysis

Statistical analyses were performed using IBM SPSS software version 24. Independent t-tests were used for comparisons between control and hypoxia in Fig. 1. In Figs. 2, 3 and 4, 2-way ANOVAs were used to compare control, hypoxia, BCH, and BCH + hypoxia groups. Multiple comparisons were corrected for using a Bonferroni post hoc adjustment. The relevant statistical descriptors for each comparison are noted in each figure legend and in Tables 2 and 3. Heatmaps and Principal Components Analysis (PCA) were carried out and plotted using ClustVis^54^. SVD with imputation was used for PCA.

## Data Availability

Data are available on reasonable request to the corresponding author.

## References

[1] Gale C (2018). Neonatal brain injuries in England: Population-based incidence derived from routinely recorded clinical data held in the National Neonatal Research Database. Arch. Dis. Child. Fetal Neonatal Ed..

[2] Lu Q, Harris VA, Kumar S, Mansour HM, Black SM (2015). Autophagy in neonatal hypoxia ischemic brain is associated with oxidative stress. Redox Biol..

[3] Gopagondanahalli KR (2016). Preterm hypoxic-ischemic encephalopathy. Front. Pediatr..

[4] Odd DE, Lewis G, Whitelaw A, Gunnell D (2009). Resuscitation at birth and cognition at 8 years of age: A cohort study. Lancet (London, England).

[5] Jacobs SE (2013). Cooling for newborns with hypoxic ischaemic encephalopathy. Cochrane Database Syst. Rev..

[6] Azzopardi D (2014). Effects of hypothermia for perinatal asphyxia on childhood outcomes. N. Engl. J. Med..

[7] Tagin MA, Woolcott CG, Vincer MJ, Whyte RK, Stinson DA (2012). Hypothermia for neonatal hypoxic ischemic encephalopathy. Arch. Pediatr. Adolesc. Med..

[8] Laptook AR (2017). Effect of therapeutic hypothermia initiated after 6 hours of age on death or disability among newborns with hypoxic-ischemic encephalopathy: A randomized clinical trial. JAMA.

[9] Mitra S (2019). Proton magnetic resonance spectroscopy lactate/N-acetylaspartate within 2 weeks of birth accurately predicts 2-year motor, cognitive and language outcomes in neonatal encephalopathy after therapeutic hypothermia. Arch. Dis. Child. Fetal Neonatal Ed..

[10] Mitra S, Bale G, Meek J, Tachtsidis I, Robertson NJ (2020). Cerebral near infrared spectroscopy monitoring in term infants with hypoxic ischemic encephalopathy: A systematic review. Front. Neurol..

[11] Bale G (2019). Oxygen dependency of mitochondrial metabolism indicates outcome of newborn brain injury. J. Cereb. Blood Flow Metab..

[12] Nijboer CH (2010). Inhibition of the JNK/AP-1 pathway reduces neuronal death and improves behavioral outcome after neonatal hypoxic-ischemic brain injury. Brain. Behav. Immun..

[13] Alexander M, Garbus H, Smith AL, Rosenkrantz TS, Fitch RH (2014). Behavioral and histological outcomes following neonatal HI injury in a preterm (P3) and term (P7) rodent model. Behav. Brain Res..

[14] Arteni NS, Salgueiro J, Torres I, Achaval M, Netto CA (2003). Neonatal cerebral hypoxia-ischemia causes lateralized memory impairments in the adult rat. Brain Res..

[15] Rice JE, Vannucci RC, Brierley JB (1981). The influence of immaturity on hypoxic-ischemic brain damage in the rat. Ann. Neurol..

[16] Neinast MD (2019). Quantitative analysis of the whole-body metabolic fate of branched-chain amino acids. Cell Metab..

[17] Steiner P (2019). Brain fuel utilization in the developing brain. Ann. Nutr. Metab..

[18] Ivanov A, Mukhtarov M, Bregestovski P, Zilberter Y (2011). Lactate Effectively covers energy demands during neuronal network activity in neonatal hippocampal slices. Front. Neuroenerget..

[19] Cotter DG, d’Avignon DA, Wentz AE, Weber ML, Crawford PA (2011). Obligate role for ketone body oxidation in neonatal metabolic homeostasis. J. Biol. Chem..

[20] Solberg R (2010). Metabolomic analyses of plasma reveals new insights into asphyxia and resuscitation in pigs. PLoS ONE.

[21] Takenouchi T (2015). Therapeutic hypothermia achieves neuroprotection via a decrease in acetylcholine with a concurrent increase in carnitine in the neonatal hypoxia-ischemia. J. Cereb. Blood Flow Metab..

[22] Solberg R (2016). Changes of the plasma metabolome of newly born piglets subjected to postnatal hypoxia and resuscitation with air. Pediatr. Res..

[23] Zhang B (2020). Regulation of branched-chain amino acid metabolism by hypoxia-inducible factor in glioblastoma. Cell. Mol. Life Sci..

[24] Li J-T (2020). BCAT2-mediated BCAA catabolism is critical for development of pancreatic ductal adenocarcinoma. Nat. Cell Biol..

[25] Daniel SK, Sullivan KM, Labadie KP, Pillarisetty VG (2019). Hypoxia as a barrier to immunotherapy in pancreatic adenocarcinoma. Clin. Transl. Med..

[26] Blackburn PR (2017). Maple syrup urine disease: Mechanisms and management. Appl. Clin. Genet..

[27] Yan R, Zhao X, Lei J, Zhou Q (2019). Structure of the human LAT1–4F2hc heteromeric amino acid transporter complex. Nature.

[28] Elorza A (2012). HIF2α Acts as an mTORC1 Activator through the Amino Acid Carrier SLC7A5. Mol. Cell.

[29] Onishi Y (2019). Hypoxia affects Slc7a5 expression through HIF-2α in differentiated neuronal cells. FEBS Open Biol..

[30] Kucharzewska P, Christianson HC, Belting M (2015). Global profiling of metabolic adaptation to hypoxic stress in human glioblastoma cells. PLoS ONE.

[31] Tiklová K (2019). Single-cell RNA sequencing reveals midbrain dopamine neuron diversity emerging during mouse brain development. Nat. Commun..

[32] Tărlungeanu DC (2016). Impaired amino acid transport at the blood brain barrier is a cause of autism spectrum disorder. Cell.

[33] Cascio L (2020). Abnormalities in the genes that encode large amino acid transporters increase the risk of autism spectrum disorder. Mol. Genet. Genom. Med..

[34] Neinast M, Murashige D, Arany Z (2019). Branched chain amino acids. Annu. Rev. Physiol..

[35] Frezza C (2011). Metabolic profiling of hypoxic cells revealed a catabolic signature required for cell survival. PLoS ONE.

[36] Utsunomiya-Tate N, Endou H, Kanai Y (1996). Cloning and functional characterization of a system ASC-like Na+-dependent neutral amino acid transporter. J. Biol. Chem..

[37] Baird FE (2009). Tertiary active transport of amino acids reconstituted by coexpression of System A and L transporters in *Xenopus* oocytes. Am. J. Physiol. Metab..

[38] Lee P, Chandel NS, Simon MC (2020). Cellular adaptation to hypoxia through hypoxia inducible factors and beyond. Nat. Rev. Mol. Cell Biol..

[39] Vannucci RC, Vannucci SJ (2005). Perinatal hypoxic-ischemic brain damage: Evolution of an animal model. Dev. Neurosci..

[40] Wu T-W (2018). Cerebral lactate concentration in neonatal hypoxic-ischemic encephalopathy: In relation to time, characteristic of injury, and serum lactate concentration. Front. Neurol..

[41] Yang SN, Lai MC (2011). Perinatal hypoxic-ischemic encephalopathy. J. Biomed. Biotechnol..

[42] Zhang Z (2020). ASCT2 (SLC1A5)-dependent glutamine uptake is involved in the progression of head and neck squamous cell carcinoma. Br. J. Cancer.

[43] Lei J, Calvo P, Vigh R, Burd I (2018). Journey to the center of the fetal brain: Environmental exposures and autophagy. Front. Cell. Neurosci..

[44] Papadakis M (2013). Tsc1 (hamartin) confers neuroprotection against ischemia by inducing autophagy. Nat. Med..

[45] Friesema ECH (2001). Thyroid hormone transport by the heterodimeric human system L amino acid transporter. Endocrinology.

[46] Kim DK (2002). Characterization of the system L amino acid transporter in T24 human bladder carcinoma cells. Biochim. Biophys. Acta.

[47] Sinclair LV, Neyens D, Ramsay G, Taylor PM, Cantrell DA (2018). Single cell analysis of kynurenine and System L amino acid transport in T cells. Nat. Commun..

[48] Wang Q, Holst J (2015). L-type amino acid transport and cancer: targeting the mTORC1 pathway to inhibit neoplasia. Am. J. Cancer Res..

[49] Lloyd AF (2019). Central nervous system regeneration is driven by microglia necroptosis and repopulation. Nat. Neurosci..

[50] Cartier J, Piyasena C, Sparrow SA, Boardman JP, Drake AJ (2018). Alterations in glucose concentrations affect DNA methylation at *Lrg1* in an *ex vivo* rat cortical slice model of preterm brain injury. Eur. J. Neurosci..

[51] Miller SP (2005). Patterns of brain injury in term neonatal encephalopathy. J. Pediatr..

[52] Steinman KJ (2009). Neonatal watershed brain injury on magnetic resonance imaging correlates with verbal IQ at 4 years. Pediatrics.

[53] Hollinshead KER (2018). Oncogenic IDH1 mutations promote enhanced proline synthesis through PYCR1 to support the maintenance of mitochondrial redox homeostasis. Cell Rep..

[54] Metsalu T, Vilo J (2015). ClustVis: A web tool for visualizing clustering of multivariate data using Principal Component Analysis and heatmap. Nucleic Acids Res..

